# Specific Bioelectrical Impedance Vector Analysis Identifies Body Fat Reduction after a Lifestyle Intervention in Former Elite Athletes

**DOI:** 10.3390/biology10060524

**Published:** 2021-06-12

**Authors:** Francesco Campa, Catarina Nunes Matias, Catarina L. Nunes, Cristina P. Monteiro, Rubén Francisco, Filipe Jesus, Elisabetta Marini, Luís B. Sardinha, Paulo Martins, Cláudia Minderico, Analiza M. Silva

**Affiliations:** 1 Department for Life Quality Studies, Università degli Studi di Bologna, 47921 Rimini, Italy; 2 Bioperformance & Nutrition Research Unit, Bettery S.A., 2740-262 Lisbon, Portugal; catarina.matias@imetrico.com; 3 CIDEFES—Universidade Lusófona, 1749-024 Lisboa, Portugal; 4 Exercise and Health Laboratory, CIPER, Faculdade de Motricidade Humana, Universidade de Lisboa, 1499-002 Cruz Quebrada, Portugal; catnunes94@gmail.com (C.L.N.); ruben92francisco@gmail.com (R.F.); fastj96@gmail.com (F.J.); lbsardinha55@gmail.com (L.B.S.); pmartins@fmh.ulisboa.pt (P.M.); cminderico@gmail.com (C.M.); analiza.monica@gmail.com (A.M.S.); 5 Laboratory of Physiology and Biochemistry of Exercise, Faculdade de Motricidade Humana, Universidade de Lisboa, Estrada da Costa, 1499-002 Cruz Quebrada, Portugal; cmonteiro@fmh.ulisboa.pt; 6 Department of Life and Environmental Sciences, Neuroscience and Anthropology Section, University of Cagliari, 09124 Cagliari, Italy; emarini@unica.it

**Keywords:** body composition, BIVA, fat mass, weight loss

## Abstract

**Simple Summary:**

The ability of specific bioelectrical impedance vector analysis (BIVA) to classify subjects according to the percentage of fat mass has been recognized in different cross-sectional studies, but no longitudinal designs have yet been applied. The results of this investigations showed that specific BIVA can be used as a practical solution for assessing body composition management in former overweight/obese athletes. In particular, reductions in bioelectrical vector length adjusted according to the specific BIVA procedure were found to be associated with reductions in percentage of fat mass.

**Abstract:**

Background: specific bioelectrical impedance vector analysis (BIVA) has been proposed as an alternative bioimpedance method for evaluating body composition. This investigation aimed to verify the ability of specific BIVA in identifying changes in fat mass after a 16-week lifestyle program in former athletes. Methods: The 94 participants included in the Champ4life project (clinicaltrials.gov: NCT03031951) were randomized into intervention (*n* = 49) and control (*n* = 45) groups, from which 82 athletes completed the intervention (age 43.9 ± 9.2 y; body mass index 31.1 ± 4.6 kg/m^2^). Fat mass was estimated by dual-energy X-ray absorptiometry. Bioelectric resistance, reactance, phase angle, and vector length were assessed by bioelectric impedance spectroscopy, and the BIVA procedure was applied. Results: A significant (*p* < 0.05) group x time interaction for fat mass, specific resistance, reactance, and vector length was found. Fat mass and vector length significantly (*p* < 0.05) decreased in the intervention group, while no change was measured in the control group. Considering the participants as a whole group, changes in vector length were associated with changes in fat mass percentage (r^2^ = 0.246; β = 0.33; *p* < 0.001) even after adjusting for age, sex, and group (R^2^ = 0.373; β = 0.23; *p* = 0.002). Conclusions: The specific BIVA approach is suitable to track fat mass changes during an intervention program aimed to reduce body fat in former athletes.

## 1. Introduction

Body composition analysis is fundamental to understand the effect of a diet and/or exercise intervention or to follow the progress of a disease [1,2]. As proposed by Wang et al. [3], it is possible to analyze body composition on the basis of five different levels: the sum of atoms, molecules, cells, tissues, or as different body segments. In sports-related populations, the most monitored parameters belong to the molecular level [1,4], in which body mass equals the sum of fat mass (FM) and fat-free mass (FFM) that can be additionally compartmentalized into total body water, minerals, and proteins.

At the molecular level, the four-compartment model is considered the “gold standard” for the assessment of FM [5]. Given that this model combines the use of several techniques, due to the assessment of bone mineral content by dual-energy X-ray absorptiometry (DXA); total body water by isotopes dilution, bioelectrical impedance analysis (BIA), or bioelectrical impedance spectroscopy (BIS); and body volume by air displacement plethysmography, it requires considerable time and high costs, as well as highly qualified personnel. In particular, BIA refers to the measurement technology that performs measurements at a single or multiple frequency. In addition, in order to clearly distinguish multi-frequency BIA from the analysis based on Cole plots or other models for fitting impedance data over the entire frequency range, the term BIS has been used to refer to the latter [4]. Among these techniques, DXA is the one that allows for the evaluation of the widest range of variables, factoring body mass according to bi-compartmental (FM and FFM) or three-compartmental (FM, lean soft tissue, and bone mineral content) models [6,7]; however, it remains a difficult technique to apply in sports populations due to the fact that the equipment is not portable and user-friendly.

BIS is currently a widely used technique for assessing body composition in several contexts [6,8,9,10]. However, the use of prediction equations limits the precision and accuracy of this method [9,11]. Therefore, it is preferable to conduct an alternative analysis that consists of the direct interpretation of the raw bioelectrical impedance parameters (resistance (R) and reactance (Xc)) [4]. One widely used approach to evaluate the raw bioelectrical impedance parameters is bioelectrical impedance vector analysis (BIVA) [4]. This technique consists of the standardization of the raw R and Xc for the height in meters of the subject and their combined interpretation within a R-Xc graph, where they are represented as a vector [12]. Recently, the effectiveness of BIVA has been shown in tracking changes in body fluids compared with a reference method (dilution techniques) [13,14]. Though the “classic BIVA” is considered the traditional method, a variant named “specific BIVA” has been proposed [6]. This approach standardizes R and Xc for the arm, waist, and calf circumferences, as well as for the height of the subject, thus contrasting the body volume effect and becoming informative about the relative quantities, such as FM%, and no-longer-absolute ones, such as TBW. The advantage of classic and specific BIVA, with respect to the only estimation of the body composition parameters, lies in the evaluation of total body water or FM% simultaneously with the phase angle, whose value is determined by the lateral position/displacement of the vector in the R-Xc graph [15]. The bioelectric phase angle is graphically represented as the angle between impedance and the x-axis, and it reflects the relationship between intracellular and extracellular fluids [15,16]. Furthermore, BIVA allows one to evaluate the vector position in comparison with population-specific tolerance ellipses [16].

The ability of specific BIVA to classify subjects according to FM% has been recognized in different cross-sectional studies, but no longitudinal designs have yet been applied [6,16,17]. This study used the results of the participants of the Champ4life project, a lifestyle intervention aimed to reduce weight in former athletes who were inactive and had overweight or obesity. Former athletes are considered an understudied group, and there is a lack of evidence regarding this topic. Therefore, this study aimed to compare FM% changes, assessed by DXA, with vector displacements measured with the specific BIVA approach after a 16-week intervention program aimed to reduce weight and body fat in inactive former elite athletes with overweight/obesity. Since specific BIVA has already been identified as a valid classification (e.g., higher or lower body fat content) tool without providing estimates of fat mass [6,16], our hypothesis was that vector displacements could reflect changes in body fat over the intervention period.

## 2. Materials and Methods

### 2.1. Participants and Study Design

This study was part of the 1-year Champ4Life lifestyle intervention targeting former athletes who had overweight/obesity and were inactive. Participants were divided in several sports, such as martial arts (25.6%), football (14.9%), athletics (mainly sprinters and middle- and long-distance track and field) (14.9%), dancing/gymnastics (10.6%), swimming (8.5%), volleyball (9.6%), handball (5.3%), rugby (3.2%), and others (7.4%). The baseline variables did not differ significantly between groups (*p* > 0.05), except for android fat (*p* = 0.034). For more details, the study protocol and the main results for this project can be found elsewhere [18]. Using a longitudinal design, two time points were considered: a baseline (0 months) and post-program (16 weeks). Ninety-four participants were included in the main study and computer-generated randomized to an intervention group (*n* = 49) or control group (*n* = 45). From those participants, 82 (control group *n* = 41; intervention group *n* = 41) completed the 16-week lifestyle intervention. Nevertheless, only 80 participants had all the data, and the intervention and control groups were therefore represented by *n* = 40 each. Details regarding recruitment, eligibility, and intervention or regarding participant characteristics and main results were stated elsewhere [18].

The study was approved by the Ethics Committee of the Faculty of Human Kinetics, University of Lisbon (Lisbon, Portugal) (CEFMH Approval Number: 16/2016) and was conducted in accordance with the declaration of Helsinki for human studies from the World Medical Association [19]. Additionally, the present study was registered at www.clinicaltrials.gov (clinicaltrials.gov ID: NCT03031951) prior to participants’ recruitment.

### 2.2. Anthropometry

All measurements were performed on the same day (approximately 8 a.m.) after a 12-h fast. Furthermore, alcohol and stimulant beverage consumption were not allowed for at least 15 h prior to testing. Participants were weighted to the nearest 0.1 kg with a scale (Seca, Hamburg, Germany), and stature was measured to the nearest 0.1 cm with a stadiometer (Seca, Hamburg, Germany) using the standardized procedures described elsewhere [20]. BMI was calculated as body mass (kg) divided by height squared (m).

### 2.3. Dual-Energy X-ray Absorptiometry

DXA was performed according to the standard procedures recommended by the manufacturer and described elsewhere [21] on a Hologic Explorer-W, fan-beam densitometer (Hologic, Waltham, MA, USA) to obtain total whole-body FM and FFM.

### 2.4. Bioelectrical Impedance Spectroscopy

Raw data (resistance (R) and reactance (Xc)) were obtained using a bioelectrical impedance spectroscopy (BIS) analyzer (model 4200B, Xitron Technologies, San Diego, CA, USA) at a frequency of 50 kHz, as previously indicated by our group [22]. The device was calibrated using the standard control circuit supplied by the manufacturer that has a known impedance (Rz ¼ 380 Ohm 1% precision and Xc ¼ 47 Ohm 1% precision). The test-retest CV in 16 participants for R, Xc, and phase angle was 0.3%, 0.8%, and 0.9%, respectively. BIS was performed with patients lying supine with their limbs placed slightly away from their body after an overnight fast and having emptied their bladders. BIVA analysis was carried out using the specific BIVA approach, i.e., multiplying R and Xc by a correction factor (A/L), where A is the estimated cross-sectional area (or 0.45× arm area + 0.10× waist area + 0.45× calf area) and L is the length of the ‘conductor’ (1.1× height). The length of the vector (VL) was calculated as the hypotenuses of individual impedance values [15]. The phase angle was calculated as the arctangent of Xc/R*180°/π [15]. In particular, by adjusting R and Xc according to the specific BIVA approach, the length of the vector became informative for the FM% (vector elongation = FM% increase; vector shortening = FM% reduction) and the lateral displacement of the vector responded to the changes in phase angle.

### 2.5. Statistical Analysis

Descriptive statistics were applied to characterize the sample. Linear mixed models included group and time as fixed effects, with baseline values and sex as covariates, to assess primary and secondary outcomes for the impact of treatment (control vs intervention group), time (baseline—0 months; post-intervention—4 months), and treatment-by-time interaction. The covariance matrix for repeated measures within subject over time was modelled as unstructured. Model residual distributions were examined graphically and by performing the Kolmogorov–Smirnov test, and no data transformations were necessary. The paired, one-sample Hotelling’s *t^2^* test was performed to determine whether the changes in the mean group vectors (measured between the first and second time points) were significantly different from zero (null vector). The paired one-sample Hotelling’s *t*^2^ test is a multivariate extension of the Student’s *t* test for paired data in comparison of mean difference vectors. Single and multiple regression analyses were performed to evaluate the associations between changes in specific VL and FM%. In addition, after the transformation of measured vector components into bivariate Z-scores (i.e., R/H and Xc/H minus the mean and divided by the standard deviation of R/H and Xc/H calculated in the reference population), bioelectrical variables were analyzed in relation to the distribution of the reference population [17]. To assess the effect size (ES) of the significant parametric test results (*p* < 0.05), the Hedge’s ES and the Mahalanobis distance (D^2^) were calculated. Threshold values were identified for ES < 0.5 as small, ES ≤ 0.8 as medium, and for ES ≥ 0.8 as large. Statistical significance for all analyses was defined as *p* < 0.05. SPSS v. 25.0 (IBM Inc., Chicago, IL, USA) was used for all statistical calculations.

## 3. Results

Eighty participants were included in this analysis. No significant differences (*p* > 0.05) in age or BMI were found between the two groups before the intervention period. A detailed description regarding the initial recruitment and the dropouts at each time point was presented elsewhere [18].

The mean values for intervention and control group before and after the intervention, divided by sex, are presented in Table 1. The intervention was effective at reducing weight and fat mass and increasing fat-free mass in the intervention group compared with the control group (interaction time x group *p* < 0.001). For bioimpedance variables, a significant (*p* < 0.05) reduction was observed for R, Xc, and VL in the intervention group. A significant time x group interaction was also found for R, Xc, and VL (*p* < 0.05). No significant (*p* > 0.05) time effect or group by time interaction for phase angle were observed.

**Figure 1:**
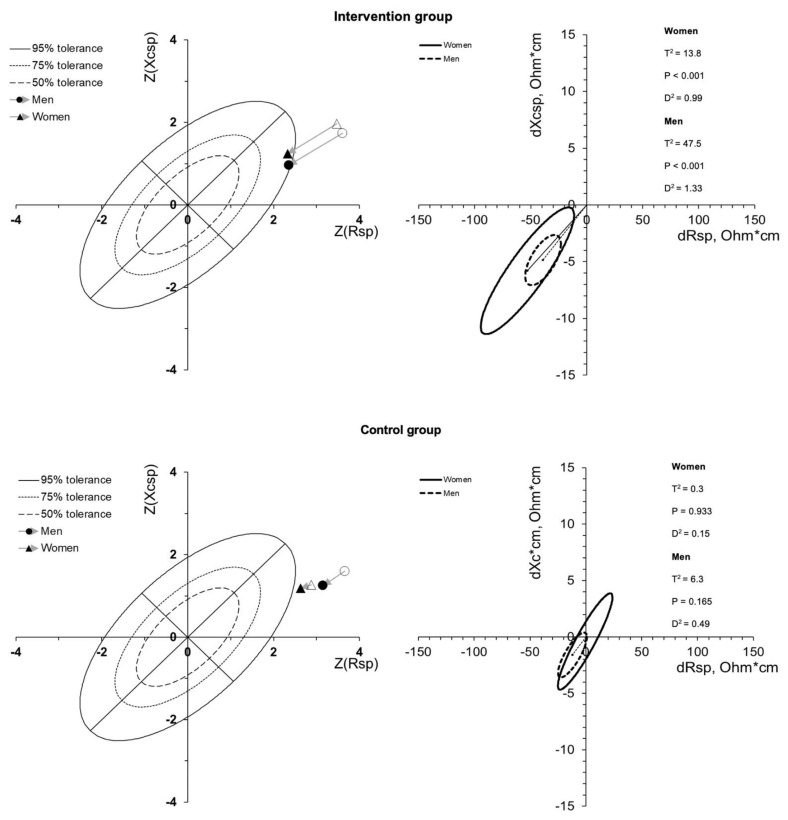
R-Xc z-score and paired graphs for the multivariate changes in bioelectrical parameters. In the left panels, bioimpedance data are plotted on the R-Xc z-score graph after the transformation of the impedance measurements from the athletes into bivariate z-scores (with respect to their reference population [16]). In the right panels, mean vector displacements with 95% confidence ellipses and results of the Hotelling’s *t*^2^ test are shown.

At baseline, the mean vectors were found to be far from the athlete-specific tolerance ellipses in both groups due to a higher FM% of the participants compared to the athletic reference population [16], as shown in Figure 1 (left panels). After the intervention period, only the group that followed the lifestyle program showed a mean vector within the tolerance ellipses (Figure 1). The paired one-sample Hotelling’s *t*^2^ test showed a significant change in the mean vector between the first (PRE) and second measurements (POST) for the intervention group (T2 = 12.9; *p* < 0.001; D2 = 1.01), while no change in the control group was assessed (T2 = 0.2; *p* = 0.920; D2 = 0.10). Figure 1 shows the vector displacements in male and female participants for both groups. Vector displacements parallel to the major axis of tolerance ellipses indicated progressive changes in FM% (higher FM% with long vectors, out of the upper pole, and lower FM% with short vectors, out of the lower pole).

**Figure 2:**
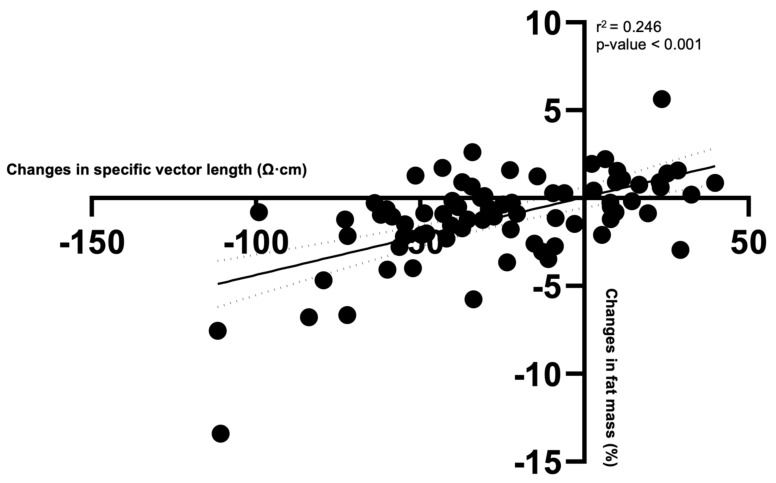
Scatter plot with changes in specific vector length and percentage of fat mass considering all participants as a whole group.

Considering the participants as a whole group, changes in specific VL were positively correlated with FM% changes (Table 2 and Figure 2), even when adjusted for age, sex, and group, as shown in Table 2. Therefore, reductions in specific VL corresponded to reductions in FM%.

## 4. Discussion

This study aimed to explore the ability of specific BIVA to assess FM% changes over a 16-week intervention program. The aforementioned intervention had a positive effect on weight, BMI, and body composition. To the best of our knowledge, this investigation, with a longitudinal randomized study design, was the first study that investigated the ability of specific BIVA to identify FM% changes in former athletes. In particular, a vector-shortening reflecting the FM% reduction observed from baseline to the second assessment moment (post intervention program) was seen in the intervention group. On the contrary, no vector displacement was observed in the R-Xc graph in the control group, which also did not reduce FM% after the 16-week period. Furthermore, a direct association between changes in specific VL and FM% was shown.

The main difference between classic and specific BIVA consists of the interpretation of the vector displacements over the longitudinal/major axis of the tolerance ellipses included into the R-Xc graph [16,23]. In fact, in classic BIVA, vector elongations reflect reduction in body fluids and vector shortening is associated with fluid accumulations [14]. On the contrary, specific vector displacements represent changes in FM% and no longer in total body water [16,24]. In this regard and in accordance with this study, the positive associations between specific VL and FM%, both in athletes and in the general population, has already been highlighted [6,16,17,24]. However, while longitudinal studies have compared changes in classic BIVA patterns with reference methods [13,14], only cross-sectional studies have investigated the relationships between specific BIVA patterns and body composition parameters derived from DXA [6,16,17]. While changes in vector length were observed in the intervention group, the phase angle remained unchanged from baseline to the second measurement. In this regard, vectors falling (steady state) or migrating (dynamic state) parallel to the minor axis and above (left) or below (right) the major axis of tolerance ellipses indicate more or less phase angle, respectively (i.e., vectors with a comparable R value and a higher or lower Xc value, respectively) [4]. As with classic BIVA, lateral vector changes along the minor axis reflect changes in phase angle and thus in fluid distribution [23]. In fact, phase angle is a good predictor of the intra/extra cellular water ratio, so we could speculate that no change in the distribution of fluids among the compartments occurred as a result of this intervention. In contrast, the results of this study showed that a shortening of the specific VL is associated with a reduction in FM% after a weight reduction program in former athletes. Therefore, the use of specific BIVA could represent a valid alternative to laboratory techniques to indirectly evaluate fat mass variations after an intervention or a training program.

The analysis of body composition nowadays represents a challenge, especially in the sports context where laboratory methods such as DXA or dilution techniques cannot always be used. For this reason, evaluation techniques such as anthropometric analysis and bioimpedance analysis are commonly performed [4]. However, these techniques present some limitations such as the need for using prediction equations to estimate body composition parameters or, in the case of anthropometry, present between- and within-operator variability [25]. In contrast, BIVA represents a qualitative approach for assessing body composition that avoids the use of predictive equations commonly used by anthropometry and conventional BIA [4]. In particular, specific BIVA appears to be a suitable method for monitoring changes in FM% in athletes. In this regard, the analysis of body fat is crucial in both former athletes and ones still engaged in sports competitions. Indeed, fat mass is considered to be a non-functional mass, with increasing amounts mechanically and metabolically hiding sports performance and negatively affecting thermoregulation, physical functioning, and general health [26,27].

A strength of this study was the randomized design and the use of DXA as a reference method. However, this study was not without limitations. In fact, DXA is not considered the state-of-the-art method for evaluating FM, but it is employed in conjunction with air plethysmography and dilution techniques in the four-compartment model, which is currently considered the gold standard. Furthermore, the results of this study cannot be generalized, but they could be applicable to assessments performed at a single frequency of 50 kHz since differences in the analysis were highlighted between bioelectrical parameters measured using single and multifrequency devices [28,29,30,31].

## 5. Conclusions

BIVA seems to be a feasible field method to assess fat mass while avoiding the use of prediction equations. The specific BIVA approach represents a suitable method for evaluating FM% and its changes following a lifestyle intervention program. In particular, vector displacements towards the lower pole of an R-Xc graph were found to reflect decreases in FM% after an intervention program aimed to reduce body fat.

## Figures and Tables

**Table 1:** Linear mixed models for the comparisons between the groups at baseline (PRE) and after 16 weeks (POST).

		Intervention (*n* 40)			Control (*n* 40)				
		Men (*n* 27)	Women (*n* 13)	Whole Sample	Men (*n* 26)	Women (*n* 14)	Whole Sample	ES ^†^	Interaction *p*-Value
**Weight (kg)**	**PRE**	95.5 ± 14.9	83.4 ± 12.0	91.5 ± 15.7	94.6 ± 17.2	76.6 ± 11.6	88.3 ± 17.6	−0.053	<0.001
	**POST**	90.3 ± 17.1	78.6 ± 11.9	86.6 ± 16.6 *	95.0 ± 18.2	76.9 ± 11.1	88.7 ± 18.4		
**BMI (kg/m^2^)**	**PRE**	31.7 ± 4.4	31.4 ± 3.5	31.6 ± 4.1	31.1 ± 5.4	29.4 ± 4.1	30.5 ± 5.0	−0.051	<0.001
	**POST**	30.2 ± 4.7	29.7 ± 3.3	30.0 ± 4.3 *	31.3 ± 5.7	29.4 ± 4.1	30.6 ± 5.2		
**FM (kg)**	**PRE**	28.0 ± 7.7	35.6 ± 6.5	30.4 ± 8.1	28.0 ± 11.3	30.7 ± 6.7	28.9 ± 9.9	0.06	<0.001
	**POST**	24.2 ± 7.8	31.9 ± 7.0	26.6 ± 8.3 *	28.6 ± 13.1	30.8 ± 6.5	29.4 ± 11.2		
**FM (%)**	**PRE**	29.1 ± 5.7	42.5 ± 3.1	33.5 ± 8.1	28.7 ± 6.4	39.8 ± 3.8	32.6 ± 7.7	0.058	<0.001
	**POST**	26.5 ± 5.4	40.2 ± 4.1	30.9 ± 8.2 *	29.0 ± 7.1	39.8 ± 3.8	32.8 ± 8.0		
**FFM (kg)**	**PRE**	67.5 ±10.9	47.8 ± 5.7	61.1 ± 13.3	66.6 ± 7.6	45.9 ± 5.9	59.4 ± 12.2	−0.093	0.054
	**POST**	66.1 ± 11.3	46.7 ± 5.2	60.0 ± 13.3 *	66.4 ± 7.4	46.1 ± 5.5	59.3 ± 11.9		
**Rsp (Ω·cm)**	**PRE**	436.7 ± 49.6	529.3 ± 40.0	466.8 ± 63.7	438.6 ± 54.1	501.2 ± 78.9	460.5 ± 69.7	0.12	0.001
	**POST**	397.6 ± 50.3	476.0 ± 71.1	423.1 ± 68.0 *	422.5 ± 57.5	489.7 ± 67.4	446.0 ± 68.4 *		
**Xcsp (Ω·cm)**	**PRE**	54.7 ± 7.1	58.7 ± 7.0	56.0 ± 7.2	53.9 ± 8.6	53.1 ± 6.8	53.6 ± 7.9	−0.105	0.009
	**POST**	49.9 ± 8.6	52.9 ± 9.2	50.8 ± 8.8 *	51.6 ± 7.5	51.4 ± 8.1	51.5 ± 7.6 *		
**VLsp (Ω·cm)**	**PRE**	440.0 ± 49.8	532.6 ± 40.0	470.1 ± 63.8	442.0 ± 54.4	504.6 ± 80.3	463.9 ± 70.4	0.123	0.001
	**POST**	400.8 ± 50.6	478.9 ± 71.5	426.2 ± 68.2 *	426.4 ± 58.8	492.4 ± 67.7	449.5 ± 69.0 *		
**PhA (º)**	**PRE**	7.2 ± 0.7	6.3 ± 0.7	6.9 ± 0.8	7.0 ± 0.8	6.1 ± 0.4	6.7 ± 0.8	−0.257	0.964
	**POST**	7.1 ± 0.9	6.3 ± 0.6	6.9 ± 0.9	7.0 ± 0.9	6.0 ± 0.6	6.7 ± 0.9		

Note: Data are expressed as mean and standard deviation.* = *p* < 0.05 vs PRE (whole sample); BMI = body mass index; FM = fat mass; FFM = fat-free mass; R_sp_ = specific resistance; Xc_sp_ = specific reactance; VL_SP_ = specific vector length; PhA = phase angle; ES = effect size; ^†^ the Hedges’ g effect size was used.

**Table 2:** Linear regression analysis for independent variables and changes (Δ) in the percentage of fat mass considering all the participants as a whole group.

Independent Variable	R^2^	SEE	β	95% CI	*p*-Value
Δ Specific vector length	0.246	2.36	0.33	0.020, 0.046	<0.001
Model 1	0.373	2.12	0.23	0.009, 0.037	0.002

Note: SEE: standard error of the estimate; β: unstandardized coefficients beta; CI: confidence interval. Model 1: adjusted for age and sex

## Data Availability

The data that support the findings of this study are available from the corresponding author upon reasonable request.
